# Simulating the host niche: balancing complexity and control in the experimental evolution of antibiotic resistance and pathoadaptation

**DOI:** 10.1099/mic.0.001767

**Published:** 2026-09-11

**Authors:** Juan Hernandez-Bird, Lucas A Meirelles

**Affiliations:** 1 Center for Computational and Integrative Biology, Massachusetts General Hospital, Boston, MA, USA; 2 Center for Integrated Solutions for Infectious Diseases, Broad Institute of Harvard and MIT, Cambridge, MA, USA; 3 Department of Medicine, Harvard Medical School, Boston, MA, USA

**Keywords:** antibiotic resistance, bacterial pathogenesis, experimental evolution, human microtissue models, pathoadaptation

## Abstract

The ‘ESKAPE’ pathogens cause the majority of antibiotic-resistant infections in humans, with associated mortality expected to surpass that of cancer by 2050. Outbreak strains of these pathogens often demonstrate a remarkable ability to establish infection and easily acquire novel antimicrobial resistance mechanisms. Because the expression of virulence factors and antimicrobial resistance genes often imposes a fitness cost, successful host-adapted strains must evolve without compromising their ability to colonize the niches found in the human body. The ongoing spread of these multidrug-resistant strains suggests that these bacterial pathogens are actively adapting to antibiotic-treated hosts. With whole-genome sequencing, we can now identify the multiple genetic changes associated with host adaptation and increased antibiotic resistance. Yet, pinpointing the specific mutations responsible for phenotypic shifts through sequencing of clinical isolates remains challenging due to the high mutational load accumulated during infection. For this reason, our understanding of how pathogens evolve within specific host niches, both in the presence and absence of antibiotics, remains limited. Experimental evolution within host tissues now allows us to more accurately simulate the conditions under which antibiotic resistance and pathoadaptations emerge. In this Perspective article, we evaluate some of the systems currently employed, discuss their respective advantages and limitations and introduce engineered human microtissue models as a promising platform for bacterial experimental evolution.

## Introduction

Since the discovery of penicillin, the development of antibiotics has transformed modern medicine. Beyond their use in treating infections, antibiotics have increased the efficacy of surgery, chemotherapy and a wide array of life-saving treatments over the last century. However, the systemic use and misuse of these ‘miracle drugs’ have accelerated the rise and spread of antibiotic-resistant bacteria, which now represent a critical threat to healthcare globally. Central to this crisis is the ESKAPE group of pathogens, comprising *Enterococcus faecium*, *Staphylococcus aureus*, *Klebsiella pneumoniae*, *Acinetobacter baumannii*, *Pseudomonas aeruginosa* and *Enterobacter* species. Together, these pathogens account for the majority of antibiotic-resistant infections worldwide [1]. For this reason, understanding the evolutionary trajectories of these organisms within clinically relevant settings is essential for developing novel strategies to counter their threat.

In this Perspective article, we examine the evolution of antibiotic resistance and pathoadaptation, highlighting how experimental evolution can simulate the environment in which these survival traits are selected for. We further evaluate the advantages and limitations of existing models used to simulate these phenomena and introduce human microtissue models as the next frontier for studying the evolution of resistance and pathogen adaptation.

## Evolution of antibiotic resistance comes at a cost

The evolution of antibiotic resistance is determined by genetic changes that increase a bacterium’s minimum inhibitory concentration (MIC) for a given antimicrobial. By definition, a pathogen is considered resistant when its MIC exceeds established clinical breakpoints, which increases the chances of ineffective treatment [2]. Resistance is often mediated by antibiotic resistance genes (ARGs), which encode enzymes that can inactivate drugs, modify or protect cellular targets or reduce intracellular drug concentrations through efflux [3].

Horizontal gene transfer of ARGs is the primary force driving the global spread of resistance [4]. ARGs are often transferred on mobile genetic elements (e.g. plasmids, genomic islands, integrons and transposons) and can move between bacteria via transformation, conjugation, transduction or uptake of outer membrane vesicles [5]. However, in the absence of horizontal gene transfer, resistance can also emerge *de novo* through spontaneous chromosomal mutations and genomic rearrangements [6]. While point mutations typically arise from unfixed errors during DNA replication, structural rearrangements (e.g. insertions and duplications) often occur at higher rates and are frequently facilitated by resident mobile genetic elements [7].

The fitness cost associated with horizontally acquired resistance is typically relatively low. One likely reason for these low fitness costs is the continuous evolutionary refinement that these elements undergo as they circulate among diverse hosts [8]. In contrast, most spontaneous resistance mutations are initially detrimental. For example, because antibiotic targets are often essential cellular components, structural or functional alterations to these targets typically impair growth in the absence of the drug [9]. While mutations are traditionally viewed as a stochastic process, recent evidence also suggests occasional strong biases [10]. Another factor modulating mutation rates is environmental stress, which often accelerates them [11]. Interestingly, in some cases, such stress can also reduce the average deleterious effects of these mutations on the organism [11]. Within an evolving population, these mutants reach fixation (i.e. high frequency) through natural selection or via population bottlenecks that prune genetic diversity [10].

A critical framework for understanding this process is the mutant selection window (MSW), which selectively enriches resistant mutants. The MSW is defined as the antibiotic concentration range between the MIC and the higher mutant prevention concentration (MPC). Below the MIC, both susceptible and resistant bacteria can proliferate, while above the MPC (typically several-fold higher than the MIC), first-step resistant mutants are suppressed. However, within the MSW, susceptible cells are inhibited, while resistant subpopulations retain a competitive growth advantage. As a consequence, prolonged exposure to drug concentrations within the MSW (e.g. due to suboptimal dosing) is a primary driver of resistance emergence [12]. Furthermore, mutations that enhance survival without strictly increasing the MIC have emerged as an important stepping stone towards the evolution of antibiotic resistance. Such mutations can promote antibiotic tolerance and persistence, allowing larger populations to survive treatment compared to the susceptible ancestor [13]. To avoid ambiguity, given that this article discusses mutations that influence both antibiotic persistence and pathogen persistence within a host, we will refer to antibiotic persistence as ‘heterotolerance’ [14].

Despite these insights, most of our understanding of bacterial evolution comes from studies performed in simplified laboratory media. These conditions mostly fail to capture many of the selective pressures pathogens encounter *in vivo*. For example, during infection, pathogens face severe population bottlenecks that limit mutational diversity [10]. In addition, they must navigate simultaneous waves of selection from recruited immune effectors and fluctuating antibiotic concentrations [15]. The immune response, in particular, is a key architect of pathogen adaptation because it drastically reduces bacterial burdens and alters the selective landscape [15, 16]. Moreover, unlike homogenous laboratory broth, host tissues comprise heterogeneous, nutrient-restricted niches where pathogens interact with host cells as free-living cells or spatially structured biofilms. To thrive in these environments, a pathogen must acquire resistance mutations that do not compromise its ability to colonize the host [6].

How do pathogens balance resistance and fitness within a host? A central question in the field regards whether this is achieved through the selection of low-cost mutations or high-cost resistance mechanisms compensated by secondary mutations. Compensatory mutations, also known as suppressor mutations, reduce the fitness costs of antibiotic resistance. These have been observed in drug-resistant clinical isolates and are believed to be the central explanation for why pathogens retain their high-cost resistance mechanisms [17]. A well-documented example of this phenomenon is the evolution of rifampicin resistance in both *Mycobacterium tuberculosis* and *P. aeruginosa*. Both pathogens incur high fitness costs when rifampicin resistance arises from mutations in *rpoB*, which encodes an RNA polymerase subunit targeted by the drug. Compensatory mutations in *rpoC* and other genes improve the fitness of these pathogens, allowing drug-resistant mutants to persist in the population after antibiotic selection is withdrawn [18, 19]. Beyond resistance conferred by chromosomal mutations, compensatory mutations have also been observed in cases of resistance, resulting from genome rearrangements and plasmid acquisition [20, 21]. The observation and validation of these evolutionary trajectories were made possible by advances in whole-genome sequencing, which has become an approach essential for studying the evolution of bacteria grown within animal tissues [16].

## Pathoadaptation: improving the ability to colonize a host niche

The evolution of pathogenesis is traditionally linked with the acquisition of virulence factors via horizontal gene transfer. These ‘gain-of-function’ traits are typically carried on plasmids or integrated into the chromosome within pathogenicity islands. While the mechanisms by which these factors promote disease are well characterized in model organisms, their production can still be metabolically expensive [22]. For this reason, pathogens can employ bet-hedging strategies, in which only a subpopulation expresses these factors during infection to preserve overall population fitness, as is the case with the production of a Type III secretion system by *Salmonella* Typhimurium [23, 24].

Beyond these major acquisitions, once in the host, the pathogen’s core genome undergoes continuous refinement to suit specific tissue conditions. This process is termed ‘pathoadaptation’. Unlike the initial acquisition of virulence genes, pathoadaptation typically involves change-of-function mutations that optimize a pathogen’s ability to thrive within a specific host niche [25]. *Yersinia pestis* provides a classic example of this two-step phenomenon. It diverged from *Yersinia pseudotuberculosis* first by acquiring two plasmids encoding virulence factors that promoted initial adaptation to the flea host. Then, subsequent genome rearrangements and gene loss further refined its adaptation to both flea and mammalian hosts, eventually enabling systemic infection [26, 27].

Interestingly, the production and assembly of virulence factors might not always come at a fitness cost. For example, constitutive expression of the Type VI secretion system in some strains of *Vibrio cholerae*, *Escherichia coli* and *Bacteroides fragilis* does not impose a significant fitness cost under certain growth conditions [28–30]. In conditions where losing this virulence factor is advantageous, its retention appears to depend on the composition of the microbiota [30, 31]. Therefore, the determinants controlling these fitness effects are likely multifactorial, including ecological interactions between microbes inside and (in cases of opportunistic pathogens) outside of the host, as well as compensatory mutations acquired after the acquisition of certain virulence factors.

### The dynamics of selection *in vivo*

The selection of pathoadaptive mutations *in vivo* is governed by shifting fitness landscapes within dynamic, spatially structured niches shaped by the microbiota, immune pressures, nutrient limitations and tissue-specific conditions [32, 33]. The dynamics of pathoadaptive mutations vary with the strength of selective pressure. For instance, mutations that confer resistance to host immune effectors may undergo rapid selective sweeps, similar to antibiotic resistance mutations, as immune cells infiltrate tissues [15]. Antigenic variation, the ability of a microbe to alter the structures recognized by the host’s immune system, is a common mechanism employed by microbial pathogens to evade clearance [34]. In contrast, mutations that subtly improve metabolic efficiency or growth rates may follow dynamics more closely resembling those observed in the Lenski long-term evolution experiment, in which it can take hundreds of generations for beneficial mutants to reach high frequencies [35]. We therefore speculate that mutations altering colonization dynamics, such as immune evasion, should achieve fixation in the host tissue more rapidly than those that optimize growth rates. Nevertheless, the fitness costs of these pathoadaptive mutations will determine whether these variants persist in the population as selective pressures wane. As with evolved antibiotic resistance, both reversion to the ancestral phenotype and the acquisition of compensatory mutations are likely evolutionary pathways in changing host environments [36].

In general, pathoadaptive mutations increase a pathogen’s ability to colonize and survive within host tissues (i.e. persistence) while paradoxically reducing the lethality of the infection (i.e. virulence). This observation supports the ‘adaptive trade-off theory’, which argues that pathogens will evolve reduced virulence when their survival depends on the host’s well-being [33]. Furthermore, a recurring theme in pathoadaptation is that improved colonization of a specific niche comes at a cost to environmental survival and transmission [32]. Therefore, for opportunistic species, pathoadaptation often represents a fundamental transition from a generalist to a specialist.

### Identifying pathoadaptive drivers

Pathoadaptive mutations can be identified by observing the independent emergence of similar mutations (or mutations that lead to a similar phenotype) across different sequenced clinical isolates. Identifying these changes requires access to patient samples from the earliest stages of infection or during documented hospital outbreaks [37]. For example, the high incidence of chronic infections in cystic fibrosis (CF) patients by *P. aeruginosa*, along with the patient’s frequent hospital visits and sample collections, has enabled the identification of pathoadaptive mutations specific to the CF lung [38–42]. In this context, *P. aeruginosa* generally reduces virulence factor production and favours a biofilm-associated lifestyle [40]. These patients frequently receive antimicrobial therapy, which acts as a powerful selective pressure guiding the pathogen’s evolution. However, it remains difficult to distinguish whether these mutations specifically promote lung colonization, reduce susceptibility to antibiotic treatment or both.

Because pathoadaptations tend to be ‘loss-of-function’ mutations, genetic screens like transposon-insertion sequencing (Tn-seq) [43], which involves genome-wide disruption of non-essential genes, can be employed to uncover novel pathoadaptive genes. In these screens, genes whose disruption decreases fitness in a host condition are identified as candidate colonization factors, while those with increased fitness may be considered candidates for pathoadaptive genes. Thus, existing Tn-seq datasets from host-tissue experiments could be mined to identify putative pathoadaptive genes [44]. Furthermore, performing Tn-seq experiments in tissues, with and without antibiotic treatment, can provide clues about mutations likely to play a role in pathoadaptation, antibiotic survival or both [45]. The ultimate validation of these candidates involves assessing whether similar genetic signatures, such as mutations causing candidate-gene knockouts, are common in real-world clinical isolates.

## Experimental evolution: observing adaptations in real time

The ability of bacterial pathogens to acquire ARGs, such as *β*-lactamases, and virulence factors, such as secretion systems, through horizontal gene transfer plays a major role in their spread. These events can be readily detected using traditional PCR methods and whole-genome sequencing techniques [46]. In contrast, elucidating how chromosomal mutations and genome rearrangements drive the evolution of bacterial pathogens is much more difficult. While access to large collections of sequenced clinical samples has enabled the identification of putative pathoadaptive genes in both pathogenic organisms and gut commensals [38, 47–51], deconvoluting the role of individual mutations remains difficult, as clinical isolates tend to accumulate numerous mutations, and the genetic manipulation of these strains is often challenging.

Experimental evolution is a research methodology that uses controlled, real-time laboratory experiments to observe evolutionary changes in populations across multiple generations. It is a powerful tool because it enables scientists to simulate specific selection scenarios, track phenotypic changes in bacterial populations and directly connect these changes to genetic modification [52]. The most prominent of these studies is the long-term evolution experiment started by Richard Lenski in 1988, which has tracked the evolution of *E. coli* in laboratory media for over 80,000 generations. These experiments have led to several landmark findings, including the observation of continuous evolution towards improved fitness in culture media, the rare evolution of citrate metabolism under oxygen-replete conditions and the emergence of hypermutator strains [53]. While experiments in simple cultures have been instrumental in improving our understanding of bacterial evolution, these systems must become more complex to account for the distinct stresses that pathogens face within a host [54]. Advances in sequencing technology, combined with the availability of animal and human *ex vivo* infection models, provide the necessary tools to better simulate the host environment [55]. Given the existential threat posed by the ESKAPE pathogens, these tools should be used to study the specific mutational pathways that drive persistent, antibiotic-resistant infections.

In the following sections, we describe several strategies for performing experimental evolution. We focus on methodologies within our expertise and highlight considerations that, in our view, should be carefully weighed when leveraging these approaches to advance our understanding of pathogen adaptation.

## Increasing the complexity of *in vitro* models

In this article, we define ‘*in vitro*’ models as experimental evolution systems where pathogens are grown in laboratory media. While these systems provide controlled environments, several factors can limit the clinical relevance of their findings. For instance, in traditional homogeneous cultures, mutant selection is primarily driven by the specific antibiotic administered [56]. However, beyond the drug itself, the physical context in which bacteria evolve may be equally consequential in determining which resistance mutations emerge and their eventual impact in a clinical setting.

The association between biofilm growth and antibiotic recalcitrance is well documented, and this lifestyle is frequently linked to clinical treatment failure [57]. Studies have demonstrated that pathogens grown as biofilms during antibiotic treatment exhibit distinct mutational profiles compared with those grown in planktonic cultures [58, 59]. In *A. baumannii*, for example, mutations that evolved in biofilms exposed to ciprofloxacin treatment more closely resembled those that arose in mice infected with the same strain and treated with the same drug [58, 60]. This suggests that performing experimental evolution in biofilms better simulates the conditions pathogens encounter during infection, leading to more clinically relevant mutational pathways. For a comprehensive review of the models and insights gained from biofilm experimental evolution, see Steenackers *et al*. [61].

Beyond physical structure, the use of tissue-mimetic media has been suggested as a simple yet effective way to simulate the host environment *in vitro. P. aeruginosa* evolved in media mimicking the CF lung more accurately recapitulates the hallmark pathoadaptations observed in clinical isolates, such as reduced motility and increased biofilm formation [62]. In this study, the authors observed that mutants with increased resistance to ciprofloxacin and ceftazidime emerged under these conditions in the absence of antibiotic selection, suggesting that reduced antibiotic susceptibility can emerge as a byproduct of adaptation to CF-like lung conditions.

Pathogens are rarely the only microbes present during an infection, and interspecies interactions can significantly alter the evolution of resistance [63]. For instance, pyocyanin, a metabolite secreted by *P. aeruginosa*, has been shown to favour the selection of antibiotic-resistant mutants in *Burkholderia* species [64]. Several other secreted metabolites can modulate antibiotic susceptibility and potentially alter the evolution of resistance [65]. Thus, incorporating components that more closely simulate the environment that pathogens encounter within the host improves the clinical relevance of findings from experimental evolution.

### Advantages and limitations of *in vitro* systems

*In vitro* systems are popular due to several key advantages. They allow precise experimental control over many conditions, including nutrient levels, temperature and population size. Furthermore, performing multiple replicates in parallel is both feasible and cost-effective. The ability to cryopreserve bacterial stocks allows researchers to ‘go back in time’ to troubleshoot contamination or to perform evolutionary replay experiments [35]. In these systems, one can easily measure fitness costs and changes in susceptibility within the exact environment in which the bacteria evolved [58]. This helps determine whether a selective advantage is specific to a lifestyle, such as biofilms, or to a particular growth medium. Using genetically tractable, laboratory-adapted strains also allows researchers to disentangle the impact of individual mutations.

However, these systems also have notable limitations. Because antibiotics are often the primary selective pressure, resistant mutants with low host-associated fitness can emerge. These mutants may be unable to compete with a susceptible ancestor in a natural host, reducing their clinical relevance. A prime example is lipooligosaccharide-deficient *A. baumannii* mutants, which can attain high levels of resistance to antimicrobial peptides when selected for *in vitro,* but the high fitness cost they incur makes this organism avirulent [66, 67]. Furthermore, the antibiotic concentrations used *in vitro* often differ from the fluctuating spatial gradients and pharmacokinetics encountered during a human infection [16]. This limitation could be addressed by using the hollow fibre infection model, which is the current gold standard for *in vitro* simulation of pharmacokinetics and pharmacodynamics. In this system, bacteria are grown behind a semi-permeable membrane through which a pump delivers antibiotics at a rate that mimics human drug levels over time, while keeping the bacteria confined for sampling throughout the experiment [68, 69]. Nevertheless, pathoadaptive mutations that reduce antibiotic susceptibility by exploiting the unique features of host tissues are far less likely to emerge in these simplified systems.

## Applying mouse infection models for experimental evolution

Mouse models are an attractive platform for experimental evolution because they provide the comprehensive features of a mammalian host, and various factors can be modulated through genetic or chemical manipulation. Within-host evolution in the mouse gut is perhaps the most accessible of these systems. Bacteria can be continuously monitored by processing faecal samples without terminating the infection. This model has been used to characterize the roles of the microbiota, host age and both innate and adaptive immune responses in pathogen evolution [70–76]. Such studies have observed parallel evolution of multiple mutations in specific genes, marking them as strong candidates for pathoadaptation to the gastrointestinal niche. For a detailed review of these findings, see Barreto and Gordo [77].

A particularly relevant study performed experimental evolution of *E. coli* in germ-free mice under cefepime treatment. The authors identified capsule mutants that survived antibiotic treatment not by increasing their resistance, but by improving their antibiotic heterotolerance and their ability to reside within host cells [78]. Loss-of-function mutations in capsule biosynthesis genes appear to be pathoadaptive across multiple species, promoting host-cell invasion and niche-specific survival [79]. Furthermore, the gut serves as a critical reservoir for ARGs due to its immense microbial diversity [80]. It has been demonstrated that *Salmonella enterica* serovar Typhimurium persisting after treatment can transfer resistance plasmids to recipient strains directly within the mouse gut [81]. These findings suggest that the mammalian gut acts not only as a strategic refuge where pathogens can evolve to survive in specialized niches but also as a dynamic hub for the horizontal transmission of resistance, complicating clinical efforts to achieve complete pathogen clearance.

The mouse respiratory tract, including the nasopharynx and lungs, also serves as a relevant model for experimental evolution. Passaging experiments with *Streptococcus pneumoniae* have identified pathoadaptive mutations specific to either the nasopharynx or the lung [82, 83]. Interestingly, adaptation to the lung often resulted in reduced virulence but enhanced persistence. In these cases, the bacteria induced less severe disease but maintained higher viable counts at the infection endpoint [83]. Therefore, inducing less severe disease in the lung niche might be more beneficial for *S. pneumoniae* in this experimental system. Similar lung passaging with *Mycobacterium canettii*, a possible environmental ancestor of *M. tuberculosis*, revealed parallel mutations that enhanced persistence by increasing resistance to oxidative stress [84]. Generally, in the absence of antibiotics, serial mouse lung passaging selects for traits that improve long-term host persistence.

Regarding the evolution of reduced antibiotic susceptibility, the incorporation of levofloxacin treatment into the *S. pneumoniae* lung passaging strategy favoured mutations that promote antibiotic tolerance rather than resistance. Target-site mutations that confer high-level resistance in *S. pneumoniae in vivo* incurred such high fitness costs that they failed to emerge even after 30 passages with increasing drug doses [85]. Indeed, a recent study expanded this finding to ten antibiotics across three host immune states. Using this strategy, the authors observed the parallel evolution of mutations in genes encoding nicotinamidase and RNase Y, which were subsequently characterized as promoting pathoadaptation and antibiotic tolerance, respectively [86]. Another group performed serial *P. aeruginosa* biofilm lung infections in the presence and absence of ciprofloxacin treatment. Under ciprofloxacin selection, mutants with increased resistance rapidly reached high frequencies, and their appearance correlated with dampened inflammatory responses in the lung [87]. These studies demonstrate that mutants selected in mammals are those whose reduced susceptibility to antibiotics does not compromise their ability to colonize a host.

Mouse models have also been used to elucidate the role of the immune system in the evolution of antibiotic resistance [60, 66, 86]. Antibiotic-treated lung passages were performed with *A. baumannii* in both immunocompetent and immune-depleted mice [60, 66]. Mathematical models suggest that the innate immune response prevents resistant mutants from reaching high frequencies [88, 89]. Indeed, during ciprofloxacin treatment, antibiotic-resistant *A. baumannii* mutants only replaced the susceptible population in immune-depleted lines. These lines exhibited a two-step mutational pathway in which mutants that increase antibiotic heterotolerance are the first to reach fixation, only to be later replaced by antibiotic-resistant mutants. An intact innate immune response blocked this pathway by preventing the initial rise of heterotolerant sub-populations [60]. In colistin-treated *A. baumannii* lung passages, antibiotic-resistant mutants reached fixation only in the immune-depleted hosts. In the immunocompetent group, a colistin heteroresistant mutant emerged that generated a colistin-resistant subpopulation, incurring a lower fitness cost compared to the resistant mutant evolved in the immune-depleted hosts. However, in this study, experimental variability precluded determining whether immune-depleted mice were more permissive to the evolution of resistance under these conditions [66].

While we are only beginning to understand these dynamics, a clear trend is emerging. Evolution within an antibiotic-treated host selects for mutations that reduce antibiotic susceptibility [60, 66, 78, 85, 86], which, in turn, facilitates the eventual emergence of high-level resistance [90]. Additionally, immunocompetent hosts may be able to suppress the evolution of *de novo* resistance in some cases. That said, this might not be the case for all pathogens, as *M. tuberculosis* has been shown to maintain greater mutational diversity when passaged in immunocompetent mice than in immunodeficient mice [91].

### Strengths and limitations of mouse models

Compared to *in vitro* approaches, experimental evolution in mice offers a higher likelihood of evolving clinically relevant mutations because pathogens encounter realistic pharmacokinetics and tissue-specific challenges. In addition, these models allow researchers to test the competitiveness of evolved strains directly in the host. In this environment, any beneficial mutation must not severely compromise the pathogen’s ability to compete with its susceptible ancestor [66, 86]. Finally, the ability to modulate host factors allows for a detailed examination of how specific immune components drive evolution.

However, mouse models also present several significant limitations. To start, compared to *in vitro* methods, mouse experiments have lower throughput, fewer replicates and higher costs. Pathogens must also survive multiple population bottlenecks during infection, which reduces genetic diversity and the probability that mutants will reach high frequencies [92]. Studies using mouse infection models often focus on acute instead of chronic infections, the latter of which are likely more relevant to the emergence of antimicrobial resistance. Furthermore, the low spatial resolution of *in vivo* systems limits the ability to determine exactly how mutations alter tissue colonization dynamics. Importantly, ethical and regulatory concerns should be considered carefully. For example, sampling a population often requires sacrificing the animal, and any optimization experiments, errors or contamination further increase the number of animals required. Finally, differences in mouse biology (e.g. physiology, immune repertoires or microbiomes) compared to humans limit the direct extrapolation of these traits to clinical settings, a limitation that can be partially overcome by employing humanized mice [93].

## Animal alternatives to mouse models

Beyond mouse models, several alternative animal hosts offer distinct advantages for experimental evolution studies, particularly when higher replicate numbers, short host generation times and reduced ethical burden are priorities. Among these, certain invertebrate models have proven especially helpful. For example, *Galleria mellonella* larvae possess an innate immune system that responds to pathogenic bacteria in ways broadly similar to those of higher animals [94]. Compared to *Caenorhabditis elegans*, which offers similar practical benefits, *G. mellonella*’s immune system is more complex, and infections can be performed at temperatures comparable to the human body [95]. Studies conducted with *S. aureus* in the *G. mellonella* model provided experimental evidence supporting that the innate immune system prevents antibiotic-resistant mutants from reaching high frequencies during antibiotic selection [96].

*Drosophila melanogaster* also has interesting potential as an experimental evolution model. Laboratory populations of this fruit fly have been selected over multiple generations for increased post-infection survival against pathogens such as *Enterococcus faecalis* and *Pseudomonas entomophila*. Such experiments revealed evolved increases in disease resistance in this host and reciprocal host–pathogen coevolutionary dynamics when the pathogen is also allowed to evolve in the host context [97, 98]. Similar to mice, this model is genetically tractable, allowing the characterization of how individual innate immune components, such as specific antimicrobial peptides, contribute to host defence during bacterial pathogenesis [99].

In addition to invertebrate models, zebrafish (*Danio rerio*) and rats are promising vertebrate alternatives to mice. Adult zebrafish can be used to study infections involving both innate and adaptive immunity, while zebrafish embryos (which possess only innate immunity) are optically transparent, allowing real-time tracking of fluorescently labelled pathogens throughout an infection. The genetic tractability of this model, combined with available chemical tools, also enables both the depletion and tracking of host effectors during infection [100, 101]. Rats, on the other hand, could also be considered an alternative murine model for experimental evolution, as some of their immune responses are more human-like than those of mice, and their greater size could support higher bacterial burdens (i.e. total c.f.u. per animal or organ) [93].

Together, these systems span a gradient of immunological complexity, generation time and experimental throughput, allowing researchers to select the model that best matches the specific evolutionary question and selective pressure under investigation.

## Leveraging human microtissue models for experimental evolution

Traditional cell culture models have also been successfully employed in experimental evolution studies of bacterial pathogens [102–107]. These models usually employ murine macrophages, as neutrophils exhibit limited viability in the lab after isolation [108]. Recent advances and the democratization of engineered organoid and human microtissue models have led to a surge in their use for studying host–pathogen interactions [109]. These systems utilize differentiated human cells to recapitulate the complex architecture and physiological defences of human tissues [109, 110]. For a detailed overview of the technical advantages and limitations of each of these models, see Alonso-Roman *et al*. [111]. While these models have been used to study human cell evolution (specifically the emergence of cancer [112, 113]), to our knowledge, they have not yet been applied to the experimental evolution of bacterial pathogens. Given their ability to recapitulate key aspects of the host niche, we propose that human microtissue models represent a powerful middle ground, offering several physiological aspects of *in vivo* systems with the experimental precision required for evolutionary studies.

In support of their physiological relevance, microtissue models have emerged as a cutting-edge platform for deconstructing host–pathogen dynamics [109, 111, 114]. Systems mimicking the airway epithelium have revealed that antibiotic recalcitrance in *P. aeruginosa* can result from localized bacterial behaviours, such as increased biofilm formation at the mucosa [45] or increased host-cell association [115–118]. Meanwhile, human colonic organoids have been used to study how the pathogen *E. faecalis* colonizes intestinal mucus [119]. In engineered models of the human bladder, tissue association and invasion have been shown to promote antibiotic survival of uropathogenic *E. coli* [120–122]. Lastly, a human epidermal model has been developed to test the efficacy of novel antimicrobials against biofilm-dwelling *S. aureus* infecting burned skin [123]. Together, these studies identify shifts in bacterial metabolism and colonization strategies, such as intracellular survival, that directly modulate antibiotic susceptibility. By using microtissue platforms, researchers may better characterize how specific host niches promote pathogen persistence and the emergence of resistance.

### Advantages and opportunities of human microtissue systems

**Fig. 1.**
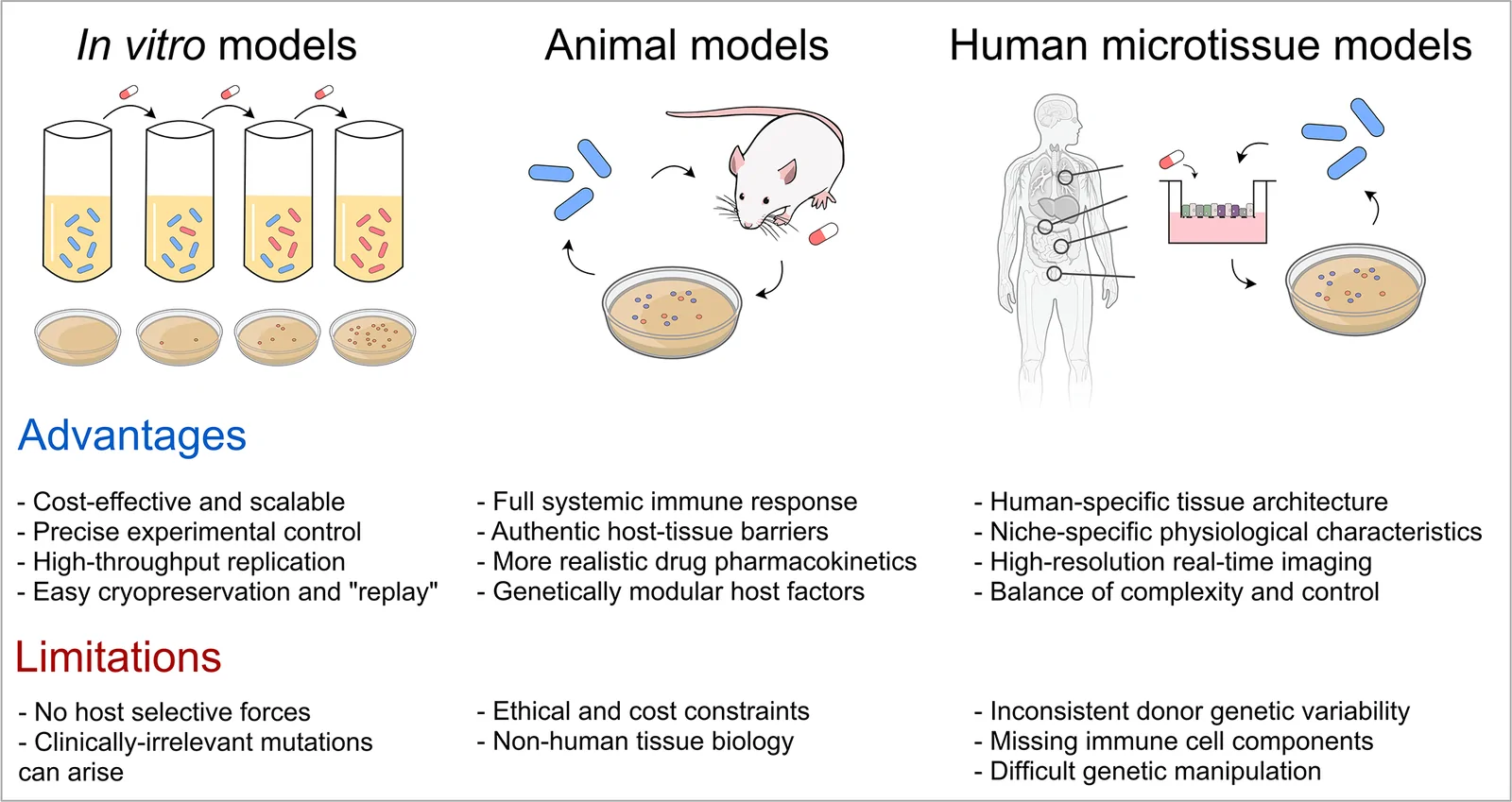
Experimental evolution systems and their advantages and limitations. Susceptible bacteria (blue rods) undergo serial passaging in various models under antibiotic treatment (pill), leading to the emergence of antibiotic-resistant mutants (red rods). The complexity of *in vitro* systems, in which bacteria are grown in laboratory media, can be increased by employing biofilm models, tissue-mimetic media and hollow-fibre infection models. Experimental evolution has been successfully performed in antibiotic-treated mouse models of pneumonia and gastrointestinal infection. Alternative animal models, with their respective advantages and limitations, are also available, as we discuss in the text. Finally, human primary cells can be harvested from various tissue sites across the body and differentiated *ex vivo* to establish microtissue models that could be used for experimental evolution. Some of the illustrations shown were modified from the National Institutes of Health (NIH) BIOART catalogue (https://bioart.niaid.nih.gov). Used illustrations are # 283, 404 and 519.

It is still early days for the use of these systems in experimental evolution. Therefore, the specific advantages and disadvantages (Fig. 1) remain partly speculative and require future experimental demonstration. Human microtissue models recapitulate many of the nutrients and cell types pathogens encounter in host tissues, as well as the chemical and physical barriers that are part of the innate immune response. Compared to animal experiments, optimization and passaging experiments can be performed at higher throughput and with more replicates, without the ethical concerns associated with animal use. In fact, current NIH guidelines advocate for reducing animal research in favour of more human-relevant models.

Furthermore, perturbing the host tissue (e.g. via injury or chemical treatment) is highly feasible in these systems and could reveal how such stressors affect a pathogen’s mutational pathways [124]. Functional genomic screens, such as Tn-seq and CRISPRi, are easier to perform in these systems than in animal models due to reduced population bottlenecks [45, 124, 125] and may provide relevant datasets while designing evolution experiments. Perhaps the greatest advantage, however, is the ability to monitor, in real time and at high resolution, how evolved mutants behave and how their colonization patterns shift within the tissue [126].

### Acknowledging challenges

While we recognize these advantages, it is also important to acknowledge the limitations and challenges of human microtissue systems. A main limitation is the challenge of integrating immune cell populations, which imposes important selective pressures that are often missing in these models [127, 128]. While efforts to incorporate immunity components are underway [129–131], the magnitude and nature of the selective pressure they exert against pathogens in these systems remain to be fully characterized. In addition, these systems traditionally required specialized expertise, although the increasing commercial availability of standardized primary cells and media is rapidly lowering the barrier to entry. Finally, the inherent genotypic and phenotypic variability of patient-derived primary cells adds another layer of biological complexity, which must be carefully considered to guarantee reproducibility in longitudinal evolution experiments.

## Choosing the right model

Ultimately, the choice of an experimental platform must be defined by the specific evolutionary question. Purely *in vitro* systems remain the gold standard for identifying the fundamental mutational landscape of resistance to novel antimicrobials. On the other hand, when investigating how systemic immunity (e.g. neutrophil or macrophage recruitment) constrains the emergence of resistant mutants, animal models remain the most appropriate choice.

However, for researchers aiming to understand how the architecture and the nutritional conditions of a specific human tissue niche influence pathoadaptation and resistance, we believe engineered microtissues offer a powerful middle ground. These platforms have the potential to uncover novel biology by revealing the complex, reciprocal interactions between evolving pathogens and the human tissues they colonize. Finally, a key point we would like to highlight is that, while we present the distinct experimental evolution models as individual approaches for comparison, only by adopting multiple systems and combining them synergistically will allow the community to dissect the complex mechanisms underpinning the evolution of antibiotic resistance and pathoadaptation. We end by highlighting some of the open questions we find intriguing and by outlining important safety precautions for performing these experiments in Box 1.

Box 1: **Open questions and safety precautions**

*Open questions*

1. In the absence of antibiotic treatment, do pathoadaptive mutations increase or decrease pathogen virulence?
2. Do distinct organs and tissue niches select for different pathoadaptive mutations?
3. Are some organs and tissue niches (e.g. the GI tract) more permissive for the within-host evolution of antibiotic resistance?
4. Does within-host adaptation occur fast enough to influence disease outcomes? Does this differ in acute versus chronic infections?
5. Does chronically diseased or damaged tissue promote the evolution of different pathoadaptations and antibiotic resistance mutations compared to otherwise healthy tissue, and if so, how?

*Safety precautions:* We urge researchers to exercise extreme caution when conducting experimental evolution with human bacterial pathogens. When selecting for antibiotic-resistant mutants, it is essential to ensure that the strain used can be treated with other clinically useful antibiotics in the event of a laboratory-acquired infection. Furthermore, if drastic increases in pathogen virulence are observed (e.g. mice show increased mortality with what used to be sublethal doses), discontinue passaging experiments with those lines and re-evaluate the safety precautions in place.

## Abbreviations

ARGs: antibiotic resistance genes
CF: cystic fibrosis
MIC: minimum inhibitory concentration
MPC: mutant prevention concentration
MSW: mutant selection window
NIH: National Institutes of Health
Tn-seq: transposon-insertion sequencing
